# Complete Genome Sequence of *Microcystis aeruginosa* NIES-2549, a Bloom-Forming Cyanobacterium from Lake Kasumigaura, Japan

**DOI:** 10.1128/genomeA.00551-15

**Published:** 2015-05-28

**Authors:** Haruyo Yamaguchi, Shigekatsu Suzuki, Yuuhiko Tanabe, Yasunori Osana, Yohei Shimura, Ken-ichiro Ishida, Masanobu Kawachi

**Affiliations:** a Center for Environmental Biology and Ecosystem Studies, National Institute for Environmental Studies, Ibaraki, Japan; b Graduate School of Life and Environmental Sciences, University of Tsukuba, Ibaraki, Japan; c Faculty of Life and Environmental Sciences, University of Tsukuba, Ibaraki, Japan; d Department of Electrical and Electronics Engineering, University of Ryukyus, Okinawa, Japan

## Abstract

*Microcystis aeruginosa* NIES-2549 is a freshwater bloom-forming cyanobacterium isolated from Lake Kasumigaura, Japan. We report the complete 4.29-Mbp genome sequence of NIES-2549 and its annotation and discuss the genetic diversity of *M. aeruginosa* strains. This is the third genome sequence of *M. aeruginosa* isolated from Lake Kasumigaura.

## GENOME ANNOUNCEMENT

*Microcystis aeruginosa* is a bloom-forming cyanobacterium in freshwater lakes and is distributed worldwide (1). A recent multilocus phylogenetic study indicated that *M. aeruginosa* is genetically divided into at least eight clades (groups A−G and X) (2). In three (groups A, B, and X), production of hepatotoxic cyanotoxins called microcystins was detected (2, 3); microcystin-producing *M. aeruginosa* causes a serious environmental problem. Seasonal succession and coexistence of toxic and nontoxic *M. aeruginosa* strains are observed in Lake Kasumigaura, Japan (2). To date, the complete genome of the toxic strain NIES-843 (group A) (4) and a draft genome of the nontoxic strain NIES-44 (group E) (5), both isolated from Lake Kasumigaura, have been determined. To characterize the genetic features of *M. aeruginosa* strains belonging to each clade and seasonal succession of bloom-forming *M. aeruginosa* in Lake Kasumigaura, further genetic information from newly isolated *M. aeruginosa* is required. Here, we report the complete genome sequence of *M. aeruginosa* NIES-2549, a nontoxic strain assigned to group G (2).

Single-cell sorting of a nonaxenic culture of *M. aeruginosa* NIES-2549 was performed using an EPICS ALTRA flow cytometer (Beckman Coulter), and the resulting axenic culture was used for DNA extraction. Sequencing of the DNA was performed on the PacBio RS II sequencer (Pacific Biosciences). A 20-kb fragmented library was constructed followed by size selection using the electrophoresis unit BluePippin (Sage Science) at 10 kb. A single library was prepared and then sequenced in two single-molecule real-time cells with P5 DNA polymerase and C3 chemistry yielding a total of 58,737 reads. *De novo* assembly was performed by the Hierarchical Genome Assembly Process (6), including assembly with the Celera assembler and polishing with Quiver. Both ends of a generated contig were overlapped across 15,000 bp. The resulting genome comprises a single circular chromosome of 4,294,213 bp with average genome coverage of approximately 123. The genome size of this strain belongs to the smallest class among previously sequenced *M. aeruginosa* genomes (5, 7).

The complete genome of NIES-2549 was annotated with the RAST server (8). The genome comprises 4,282 protein-coding sequences (CDSs), including 1,720 hypothetical proteins, 41 tRNA genes, and two sets of rRNA genes. The G+C content of the genome is 42.92%. The sequence of 16S rRNA was compared with those of NIES-843 and NIES-44, resulting in 99.66% and 99.64% similarities, respectively. The genome shares 3,342 CDSs with that of NIES-843 and 3,307 CDSs with that of NIES-44. In the NIES-2549 genome, 940 and 975 unique CDSs were found when compared with those of NIES-843 and NIES-44, respectively. The gene order was compared with that of the complete genome of NIES-843 using Murasaki (9). Large numbers of inversions and transposes across the whole genome were detected between two genomes; however, most genome segments were conserved as a whole. This result indicates that frequent rearrangement occurred in the *M. aeruginosa* genome.

### Nucleotide sequence accession number. 

This whole-genome shotgun project has been deposited in GenBank under the accession no. CP011304.
